# User-Centered Design for Designing and Evaluating a Prototype of a Data Collection Tool to Submit Information About Incidents of Violence Against Sex Workers: Multiple Methods Approach

**DOI:** 10.2196/53557

**Published:** 2024-10-09

**Authors:** Melissa H Ditmore, Jose Fernando Florez-Arango

**Affiliations:** 1 CUNY School of Public Health and Health Policy, 55 W 125th St, New York, NY, 10027, United States, 1 3475609159

**Keywords:** mobile health, sex worker, user-centered design methods, usability, heuristic analysis, cognitive walkthrough, aggression, abuse, occupational health, reporting, prototype, heuristics, human-centered design, implementation, barriers, enablers, data collection, digital health, underreporting

## Abstract

**Background:**

Sex workers face an epidemic of violence in the United States. However, violence against sex workers in the United States is underreported. Sex workers hesitate to report it to the police because they are frequently punished themselves; therefore, an alternative for reporting is needed.

**Objective:**

We aim to apply human-centered design methods to create and evaluate the usability of the prototype interface for ReportVASW (violence against sex worker, VASW) and identify opportunities for improvement.

**Methods:**

This study explores ways to improve the prototype of ReportVASW, with particular attention to ways to improve the data collection tool. Evaluation methods included cognitive walkthrough, system usability scale, and heuristic evaluation.

**Results:**

End users were enthusiastic about the idea of a website to document violence against sex workers. ReportVASW scored 90 on the system usability scale. The tool scored neutral on consistency, and all other responses were positive toward the app, with most being strong.

**Conclusions:**

Many opportunities to improve the interface were identified. Multiple methods identified multiple issues to address. Most changes are not overly complex, and the majority were aesthetic or minor. Further development of the ReportVASW data collection tool is worth pursuing.

## Introduction

Sex workers are frequently victimized by a variety of perpetrators [1] due to lack of empathy and because people who commit crimes against sex workers know that their crimes are unlikely to be reported. Sex workers are targets of violence, with 32% to 55% reporting violent experience in the previous year [2], but they often do not report violent incidents [2] in part because they encounter problems when they seek to report violence to police. In fact, police may arrest them for sex work instead of investigating violent crimes against sex workers; sex workers have also been victimized or harassed by police [3,4]. Those who seek care after victimization may not reveal their status as sex workers because health care providers may stigmatize and discriminate against them. These factors culminate in an unfortunate lack of knowledge in the domain. Documenting violence against sex workers is a necessary prerequisite to demonstrating the need to address interpersonal violence against sex workers, and will help develop more effective responses and raise funds for their implementation. Violence against sex workers is associated with sequelae including post traumatic stress disorder [5], HIV, and other sexually transmitted infections [6].

Opportunities to increase the likelihood of sex workers reporting violence include changing police practices such that they would accept reports and investigate violence, changing laws to decriminalize sex work or legalize sex work such that some sex work would be within the law. Legal support could help sex workers who want to report violence to police. There are at least two nonprofit organizations that offer legal services to sex workers in the United States. Sex workers share information about perpetrators of violence among themselves [7] separate from reporting to law enforcement, in order to share information about violent experiences and help others avoid their attackers; however, it is impossible to know how widely these mechanisms are used online, via text messages, or on paper. In contrast, an app was commissioned by the Asia Pacific Network of Sex Workers and designed for sex workers in Myanmar to help them report violence committed against them to the police.

The app (iMonitor+, DureTechnology) was part of a larger program, and they used the app or a hotline that connected victims of violence to a service provider who accompanied them to the police to report violence and to health care providers [8]. Most sex workers in Myanmar have smartphones, but uptake of the app was not strong, in part because they preferred using an existing hotline to the app [8]. As the app was commissioned by sex workers who are part of a regional international network, it exhibited some aspects of user-centered design (UCD) because sex workers explained what they wanted to include; moreover, the context of use was well understood and the users specified the end requirements. The app was not used to share information about perpetrators of violence. Other apps have been developed for sex workers in Cambodia [9] and South Africa [10].

The lead author (MHD) was commissioned to evaluate the antiviolence program for sex workers in Myanmar. After conducting the evaluation and learning more about the Burmese app, MHD was inspired to try to develop something such as this for use in the United States. As a result, we developed a prototype of a data collection tool that we hope sex workers would feel confident using to report violent experiences. The prototype mobile health (mHealth) data collection tool *ReportVASW* is intended for sex workers in the United States to report violence committed against them. VASW stands for violence against sex worker, and the tool collects data reported by sex workers who have been victims of violence. The interface was designed to enable multiple options for reporting violence, including drop down menus, open text, and audio recording. ReportVASW can be pronounced Report Violence.

The victimization of sex workers has been a long-term focus, and we have published multiple reports and papers about violence against sex workers and documented human rights violations in multiple locations in Africa [11], Asia [12], and the United States [13,14].

Most systems are set up to be used repeatedly if not constantly; ReportVASW is different because as it addresses violent victimization of the end user, it is hoped that most people never need to use it, and that those who do will use it once or rarely. For this reason, we believe ReportVASW should be intuitive and easy to use, without a learning curve.

## Methods

### Design Process and Prototype

UCD was used to design this prototype because only active sex workers seeing clients face-to-face can describe their current methods for sharing information about violent people and what they do to try to avoid violence in their work. Considering that the user context is in the aftermath of a violent and possibly traumatic event, ease of use is paramount, and so developing an app that is easy to learn and quick to use is important. These are hallmarks of UCD.

The aim of UCD is to improve usability by maximizing effectiveness, efficiency, and satisfaction of end users in the specific aim of the product in question. Careful application of UCD methods at the earliest stages should reduce user error, and limit cost and time spent redesigning after developing software.

UCD follows specific principles, including focusing on users and tasks, measuring usability empirically, and iterative testing of design and usability. UCD has been used with success to develop mHealth apps and health record systems [15,16]. These principles were at the heart of the specific methods used for each of the 4 steps. The steps of the UCD approach align with more specific methods; for example, contextualization using functional analysis and consulting potential end users, and ideation through task analysis focused on the end user group and what steps would be required to successfully complete the task; prototyping using representational analysis of the tool; and finally, usability testing using scenario-based testing and heuristic analysis. This study’s design process used the 4 methodological steps associated with UCD.

### Step 1 - Contextualization

While violence against sex workers has been studied, few efforts to address this violence have been evaluated in the United States [17]. The authors seek to develop a new tool to respond to violence against sex workers. Contextualization was undertaken through desk research using functional analysis and consultation with active sex workers (user analysis) to ask whether such a tool would be useful; some were interested and offered opinions on the proposed tool. In total, 4 of 5 sex workers consulted were interested in the project at least in theory. UCD with multiple methods, such as considering both end user and design use context, has been used with success in mHealth [18,19] but it is not without difficulties [20,21]. Formative research using UCD can be time consuming, and users are not always easy to engage, but the literature shows the value of engaging end users in formative processes [20,21].

### Step 2 - Ideation

The lead author thought through what this data collection tool should ask, how to collect information, and what information is most important. This process included task analysis, identifying the intended task and using a flowchart on paper to plan the way the tool would collect information.

### Step 3 - Prototyping

The lead author engaged in representation analysis using the final flowchart to inform illustrations of what the screen would look like at each step. This was followed with the lead author attempting to apply Nielsen Heuristics to assess and refine the prototypes. Not all steps needed extensive revision. The initial paper prototype included 7 screens.

The prototype was designed using the free version of Figma software online tool as of April 26, 2022, which allows the creation of 3 pages only. The heuristic analysis was based on Neilsen “10 usability heuristics.” [22]
Representational analysis consisted of heuristic analysis undertaken by the authors and 2 colleagues. The form used is a spreadsheet developed by the second author based on the work of Zhang and Wallji [23,24], in which a scale of 0‐4 is used to grade each issue, from minor (1) to catastrophic (4), and 0 used to indicate disagreement that the point is an issue. The spreadsheet also contains a column labeled “proposed solution.”

### Step 4 - Usability Testing

Multiple methods were used to evaluate the ReportVASW interface. After the prototype was developed, usability testing using representation analysis was undertaken via heuristic analysis and task based and scenario testing. Heuristic analysis of the prototype was undertaken by the authors and 2 colleagues.

Additionally, the lead author and a developer recruited a convenience sample of 5 end users who self-identify as female with experience in a variety of sex work venues (escorts, brothel workers, sadomasochism professionals, and strippers) to test the usability by entering data from scenarios provided (scenario based testing) in a cognitive walkthrough [25], using the proposed app to enter data from a scenario taken from interviews with sex workers; all 5 agreed to do the walkthrough with the paper prototype and verbal consent was obtained. Paper prototypes have been used with good results in developing and testing prototypes [26]. In total, 4 of the 5 end users recruited did the cognitive walkthrough during the spring of 2022, three in private locations and one in an office (one was not available after contracting COVID-19). The scenarios used were taken from our previous research [13,14] and are outlined in Multimedia Appendix 1.

This protocol was submitted to the CUNY Graduate School of Public Health and Health Policy Human Research Protection Program, and was classified as exempt. No incentives were offered.

A 10 item system usability scale (SUS) was brought to the second cognitive walkthrough and asked the end user to rate the 10 statements, which were read aloud (by MHD), as a back-up evaluation method. SUS is a 10-item Likert scale, with each item’s score ranging from 0 to 4. Odd numbered items are scored at the scale position minus 1 and even numbered items are scored at 5 minus the scale position; the sum of the scores is then multiplied by 2.5 to obtain the overall score [27,28].

The following sections of this paper detail the evaluation methods undertaken in usability testing with a multidisciplinary team of informatics professionals and 4 potential end users based on the known theory that this number will generally expose the majority of problems with usability [25], in our attempt to evaluate the usability of the first prototype of ReportVASW.

### Ethical Considerations

This project was deemed exempt by CUNY Graduate School of Public Health and Health Policy since it focused on the usability of a data collection tool and did not involve human participants or personal information.

## Results

### Step 1 - Contextualization

Sex workers consulted confirmed that the data collection tool should make it easy to report incidents of violence, including location, what violence occurred, and who committed this violence. They confirmed that the site should be easy for people who are in the aftermath of a traumatic event to use, demonstrating understanding of the end user population, sex workers.

Step 1 is the functional analysis of ReportVASW. The information collected using ReportVASW could be used in multiple ways:

1. This site may facilitate information sharing in addition to documenting violence committed against sex workers and generate evidence to be used in reporting violent incidents in which sex workers are victimized.
2. Geolocation data about violent incidents can be used in the allocation of resources by organizations that work with sex workers, and in advocacy for additional resources.
3. The app could connect sex workers who have been victims of violence to an organization offering services to sex workers, perhaps including trauma-informed service providers, and possibly to attorneys in the area where the crime was committed. This would need to be determined by location; local sex worker groups would be consulted about friendly services to reach out to.

### Step 2 - Ideation

**Figure 1.**
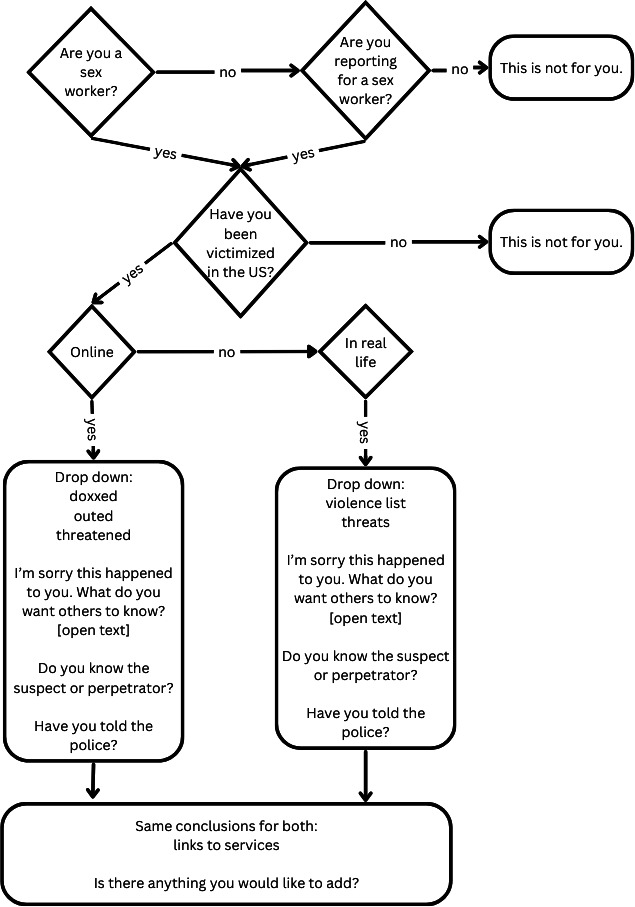
Ideation product: flowchart.

The flowchart (Figure 1) went through 3 drafts in an iterative process. During this process, the ways the information would be collected and the order of questions were changed, including adding questions, each time making adjustments to the information presentation and order and ideas about how to collect it. The task analysis aspect of ideation was fruitful, because it forced the developers to clarify what should be identified and reported. The first component in the app is screening questions about sex work experience and victimization, the second component asks about the victimization, the final component would offer links to services.

### Step 3 - Prototyping: Representational Analysis

**Figure 2.**
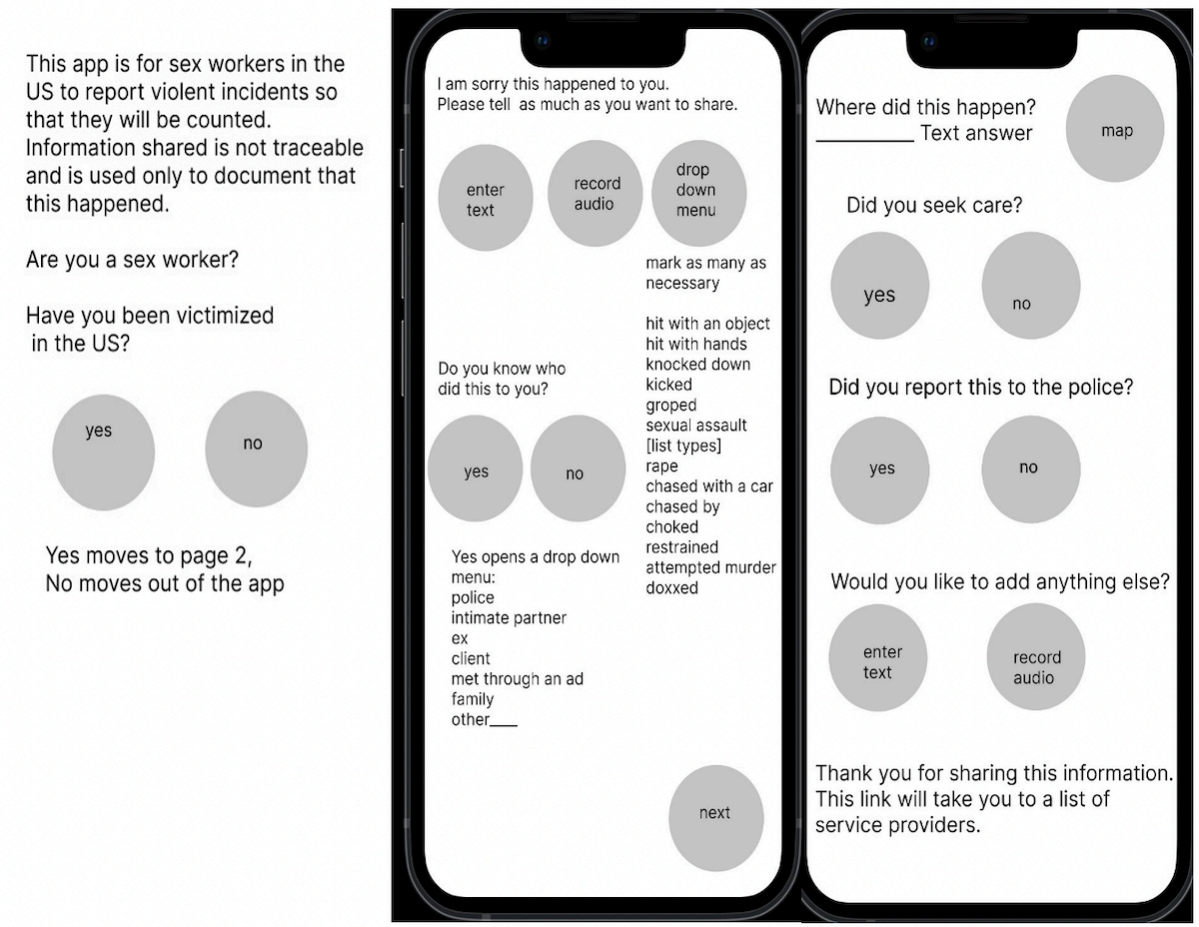
Prototype screens (developed using Figma).

The heuristic analysis and cognitive walkthrough using scenario-based testing methods generated similar assessments about the ease of use of the prototype and that the aesthetics should be improved; this overall agreement would seem to indicate that the findings reflect the actual usability of the prototype ReportVASW (Figure 2).

### Evaluation Outcomes

Responses were compiled and average scores computed; heuristic analysis scores ranged from 0.5 to 3.25 (Table 1) and revealed that most problems are aesthetic or minor. The consistency inspection was the easiest to undertake, revealing that there were multiple problems with aesthetics:

- Buttons do not line up, looks messy.
- Labels are not uniform, labels are all over the place on the buttons.
- Color scheme is the default, and can be improved.

**Table 1.** Most severe problems identified by heuristic analysis, with scores over 2.5.

Problem	Heuristic violation characteristic	Average (SD)
Aesthetics	Aesthetics	3.25 (1.50)
No way for user to know if they can erase what they wrote or said or resubmit	Undo	3.25 (0.96)
Submit button missing	Minimalism	2.75 (1.26)
Wording is too direct, recommend using words that are sensitive to user so they can feel comfortable sharing	Language	2.75 (0.50)
Map: no indication if this is based on zip code, neighborhood, city, state	Visibility	2.67 (0.50)
Hard to read because there is too much on screen	Visibility	2.5 (1.29)

These are all aesthetic and minor changes, simple to address, and not an obstacle to development. The aesthetics, however, need more attention. Consolidating questions may have compromised usability by rendering the screen too crowded because they incorporate too many frames, thereby decreasing visibility and violating the heuristic of minimalism. The default color scheme should be changed.

The number of violations of each heuristic listed in the Heuristic Analysis chart were counted (Table 2). The most frequently violated heuristics were visibility (6), consistency (4), and minimalism (4). These are related: addressing violations of the heuristics of visibility and consistency will contribute to minimizing violations of the heuristic of minimalism.

**Table 2.** Frequency of heuristic violations.

Characteristic	Value (n)
Visibility	6
Consistency	4
Minimalism	4
Language	3
Control	2
Documentation	2
Flexibility	2
Feedback	1
Undo	1

### Step 4 - Usability Testing: Scenario-Based Evaluation by Cognitive Walkthrough and Task Analysis

#### Overview

The first 2 cognitive walkthroughs and task analysis became in-depth discussions with input from the participating end user; end users confirmed the need for an application such as ReportVASW, and said that it could be a viable tool for collecting information about violence committed against sex workers, especially incidents that remain uncounted because they were not reported to police or victims did not present at hospitals, and is worth pursuing. The cognitive walkthrough process delivered positive feedback and users offered many ideas for improvements. Suggestions included new features, the collection of additional information, and aesthetic and functional comments, such as specific text for an introductory screen. Further, 4 end users completed the evaluation; the fifth was excluded because of illness.

#### Task Completion Time

In total, 2 people who completed the task did so in under 90 seconds. The other 2 end users offered more information than was asked during the cognitive walkthrough and so the timing of the actual task was not possible to measure; the discussion took over 20 minutes. All end users were very satisfied with the flexibility of multiple ways to submit information. Users felt that with a submit button, it would prove an effective way to collect information about the epidemic of violence against sex workers. End user testers of the prototype (Figure 2) made some recommendations, which are listed below.

#### Aesthetics

- Use universally recognized symbols where possible, for example, a microphone emoji for “record audio.”
- Improve attractiveness through color scheme and format; usability.
- Specify clearly that people can use any and all of these methods (text, record audio, or drop-down menu) to submit information.
- Consider 1 question per screen, which would advance without a “next” button. It may be possible to do this with multiple forms of data submission.
- Move open text field in drop-down menus to top, in order for people not to need to scroll to see it.
- Add a submit button.

#### Changes to Data Collection

- Change the screening question about victimization in order to capture data from people who do not identify as victims, but who have had violent experiences.
- Add a date field for the event, perhaps simply year, to distinguish recent events from long ago events.
- Add “drugged” to drop-down menu of types of victimization
- Change “did you seek care?” to specify “did you see a doctor or go to a hospital?” in order to be more clear, so that users will not include calling or visiting friends.
- Add branching questions after “did you report this incident to the police?” including “did they take your report?” and “were you treated respectfully?”
- Add demographic information about race and gender
- Ask whether the data should be shared with people collecting information about people sex workers should avoid (ie, a “bad date list”).

#### Additional Input

- Make more clearly anonymous, with an introductory page that emphasizes anonymity and the why of ReportVASW, emphasize that IP address and mobile numbers will not be stored, therefore “you can’t be traced or tracked” – this page must be clear, concise, and convincing.
- “Location” is ambiguous, and could be an address, as on a map, or a venue such as “car” or “brothel.” Even place names can be unclear: Springfield is a town in every state.
- Ask whether the information was shared with other sex workers, as in a “bad date list.”
- One end user tester suggested using speech to text with the audio function and enabling the speaker to edit in the moment.
- A critical point raised by the third tester was that the screening questions are good but will miss some people, highlighting that this person did not identify as someone who had been victimized, and so would be eliminated by the screening question “have you been victimized in the United States?” but had been drugged, threatened with a gun, and raped, at different times.
- A second critical point raised by the fourth tester was that the final question, “would you like to add anything else?” could lead to actionable information being shared without any way to act on this, including but not limited to suicidal ideation, violent impulses, and information about human trafficking situations. It was recommended that this open-ended question be eliminated to preclude the possibility of liability and to limit mental anguish for the person addressing these reports.
- All 4 agreed that entering the data from the scenarios was possible and easy, but nuance would lost without offering open-ended formats.
- One user pointed out that recounting violent experiences takes emotional energy, prompting her to ask, “Without a clear benefit to the victim, why do it? Can there be a way to connect to targeted services for the individual to report/record?”

### About SUS

ReportVASW scored 90 on the SUS; SUS scores over 68 are considered good [27,28] (Table 3). The tool scored neutral on consistency, and all other responses were positive toward the app, with most being strong.

**Table 3.** System usability scale chart scores for ReportVASW.

	Strongly agree	Agree	Neutral	Disagree	Strongly disagree
I would like to use this	✓	—^a^	—	—	—
It is too complex	—	—	—	—	✓
Easy to use	—	✓	—	—	—
I need IT support	—	—	—	—	✓
Functions are well integrated	✓	—	—	—	—
Too much inconsistency	—	—	✓	—	—
Most would learn it fast	✓	—	—	—	—
Cumbersome	—	—	—	✓	—
I feel confident using it	✓	—	—	—	—
Requires much learning	—	—	—	—	✓

a Not applicable.

## Discussion

### Step 1 - Contextualization

UCD with multiple methods was successfully implemented in the development and evaluation of this first prototype, reflecting both success [15,16,18] and difficulties [20,21] reported by others, with the additional aspect of an atypical end user for informatics.

### Step 2 - Ideation

Using multiple methods to evaluate the prototype enabled the collection of new information, including phrasing for screening questions, and positive reception of ReportVASW. New knowledge was gained from the evaluation, particularly through engagement with end users, even considering the lead author’s significant expertise, particularly information about additional topics and language to incorporate in the next version of the prototype. The literature reflects the usefulness of multiple methods, despite challenges [20,21].

We believe we have sufficient input and information to proceed to significantly improve the next draft of the prototype, because the end user evaluation aligned with the heuristic analysis. End user comments offered solutions to issues identified in heuristic analysis particularly regarding aesthetic and functional issues; these solutions will be applied in the next steps. Additionally, UCD was useful in evaluating the ReportVASW prototype interface because adaptation is necessary to bring something designed for an Asian context to the American context; input from end users will improve this adaptation. Evaluations of the interface using heuristic analysis and end user scenario-based testing will inform the revisions to the prototype.

### Step 3 - Prototyping: Representational Analysis (Heuristic Analysis)

Figma’s 3 screen limit encouraged the consolidation of some of the screens, thereby making the app simpler. This involved consolidated screening questions on page 1, information about a violent event on page 2, and information about seeking care and reporting and asking if there is anything more they would like to share on the final screen. This reduced number of screens may be better than the initial drawings with 1 question per screen. Each step, from the flowchart (Figure 1) to the paper draft to the digital pictures (Figure 2) offered opportunities for improvement.

The heuristic analysis forms the baseline for the evaluation of the prototype through its next iteration. This analysis offered actionable recommendations and afforded interesting discussion related to the varied backgrounds of the analysts. For example, the most technically skilled of the analysts disagreed that some things were necessary, while an analyst with experience working with different communities offered important points about sensitive language, which the lead author is confident can be addressed through consultation with end users.

### Step 4 - Usability Testing: Task Analysis by Users

As shown above, end users completed the task quickly, and offered substantive input. Their interested and substantive responses indicate a need for ReportVASW. Most information offered was concrete and included suggestions that can be easily incorporated, for example, each offered ways to formulate specific questions, about seeking care and about location. However, some of the input is not as easily addressed, such as how to phrase screening questions in order not to exclude people who do not see themselves as victimized. Other input pushes the developers to find ways to benefit participants, who are expending energy to share information about potentially traumatic experiences. Possibilities include offering a list of referrals to service providers around the United States, including clinical therapists and supportive trauma-informed health care professionals, in partnership with existing services used by sex workers. Further, 1 complication is that most services for victims of violence focus on women, usually cisgender women; however, sex workers of any gender may be victimized [1-3].

Sharing the information collected with “bad date lists” about people who commit violence against sex workers is more complicated that it sounds because of recent US legislation; law enforcement efforts have led to the closure of online venues for information sharing among sex workers [29,30]. While sex workers actively share their concerns online [31], US sex worker groups are decentralized, and sex worker groups alert their members about reports of bad dates; ultimately, ReportVASW should be managed by sex workers. Each could have copies of decentralized data, and in the future we will need to explore alternatives to manage this data, for example, using blockchain.

Adapting standardized methods to the end user population has been challenging to others, who recommend flexibility and accommodation of end users over rigidity about standardization [21]. The prompt for end users to begin the evaluation task must be chosen wisely. Cognitive walkthroughs with 2 end users featured interruptions with salient and helpful input. The third and fourth people who conducted the cognitive walkthrough each took approximately 90 seconds to complete the task. This time certainly does not account for the difficult nature of the material; none of the testers were recent victims of potentially traumatizing situations.

### Next Steps

Follow-up is essential to the findings and implications of the project. We have received actionable recommendations through the cognitive walkthrough and the heuristic analysis that indicate clear urgent next steps. The agreement between the task analysis of the cognitive walkthrough and the input from the cognitive walkthroughs and the heuristic analysis included many recommendations addressing aesthetics, usability, data collection, and other input about ways to improve uptake and also increasing end users’ confidence that ReportVASW is benign and not used for surveillance. Immediate next steps based on this input include:

- Adding all the input in changes to screening and data collection offered by end users, including adding a convincing introductory page about the use of the data and lack of tracking, as identified in heuristic analysis and with suggestions made during cognitive walkthrough,
- Making the urgent changes identified in the heuristic analysis including redesigning the interface for consistency and improving attractiveness.
- Exploring ways to link people providing input to services.

The most important next step will be to link users to services that could be helpful in the aftermath of violence, including the long-term aftermath, involving long-term effects of violence such as chronic disease [6] and post traumatic stress disorder [5]. It is not clear whether sharing links to legal and social services would meet this need. There are few low-barrier services for sex workers in the United States, presenting an obstacle to access. Considering this, it may prove beneficial to collaborate with an existing program offering legal and/or health services for sex workers. End users must be involved in the decisions about services included, in order to identify service providers that do not stigmatize or discriminate against sex workers. Additionally, geolocation data about violent events should be used to help identify where services are most urgently needed. Building more evidence will contribute to understanding reasons for sex workers to report violence against sex workers. However, police resistance to investigating violence against sex workers cannot be addressed by an app, and the data collected may be used in advocacy.

The next version of the prototype will also be evaluated using heuristic analysis. Comparing these sequential heuristic analyses will help determine priorities for changes to the following version. Using multiple methods for all 4 steps of the UCD process gave us richer information than we would have had using only 1 method at each step. While the time invested was significantly more than it might have taken using only 1 method, the benefits are great because the information gathered offers more certain next steps and reduces the chances of missing important elements that could require additional versions later.

### Limitations

Figma constrained design possibilities that contributed to more creative ways to include information in less space. The number of individual end user evaluators was in the ideal range of 4 to 5 [25], while the cognitive walkthrough might benefit from another end user because of the interest in sharing additional information. The convenience sample of end users also presents a limitation, and data could have been different from people who were not familiar with the lead author, and the sample includes only self-identified women. SUS input may have been influenced by the lead author reading the questions and asking the answers, rather than the end user checking the boxes themselves. Additionally, the creator’s bias impeded her ability to test the prototype, due to extreme familiarity. The small number of evaluators for the heuristic analysis presents a limitation.

### Conclusions

The value of using multiple methods in UCD was clearly demonstrated in the process of designing and evaluating a prototype data collection to submit information about incidents of violence against sex workers. Using multiple methods in the initial steps of contextualization and ideation led to multiple revisions in these early stages. Using multiple methods in prototyping and evaluating the prototype afforded the opportunity to collect informative input from people in different roles, including end users and informatics professionals. The results from each method aligned such that the representational analysis, the consistency inspection, and heuristic analysis reinforced ways to improve the prototype, reinforcing the input from each source. End users confirmed the need for an application such as ReportVASW and that developing the data collection tool is worth pursuing, and informatics personnel reinforced the feasibility and offered insight to improve its design and utility. The use of multiple methods to evaluate the prototype contributed to a greater understanding than any single method alone.

## Supplementary material

10.2196/53557 Multimedia Appendix 1 Scenarios used for the cognitive walkthrough.

## Abbreviations

mHealth: mobile health
SUS: system usability scale
UCD: user-centered design
VASW: violence against sex worker

## References

[1] Decker M, Rouhani S, Park JN (2020). Incidence and predictors of violence from clients, intimate partners and police in a prospective US-based cohort of women in sex work. Occup Environ Med.

[2] Deering KN, Amin A, Shoveller J (2014). A systematic review of the correlates of violence against sex workers. Am J Public Health.

[3] Stenersen MR, Thomas K, McKee S (2024). Police harassment and violence against transgender & gender diverse sex workers in the United States. J Homosex.

[4] Fehrenbacher AE, Park JN, Footer KHA, Silberzahn BE, Allen ST, Sherman SG (2020). Exposure to police and client violence among incarcerated female sex workers in Baltimore city, Maryland. Am J Public Health.

[5] Park JN, Decker MR, Bass JK (2021). Cumulative violence and PTSD symptom severity among urban street-based female sex workers. J Interpers Violence.

[6] Decker MR, Park JN, Allen ST (2020). Inconsistent condom use among female sex workers: partner-specific influences of substance use, violence, and condom coercion. AIDS Behav.

[7] CBC (2021). News KL CBC-wide “bad date” reporting system aims to improve sex worker safety. https://www.cbc.ca/news/canada/british-columbia/b-c-bad-datedatabase-sex-worker-safety-1.5897871.

[8] Ditmore M Aye myanmar association’s community-based intervention to combat violence against sex workers, 2017-2020. United Nations Trust Fund to End Violence Against Women (UN Women).

[9] Brody C, Chhoun P, Tuot S (2022). A mobile intervention to link young female entertainment workers in Cambodia to health and gender-based violence services: randomized controlled trial. J Med Internet Res.

[10] SANAC sex worker program. DureTechnology.

[11] (2013). Population size estimation of key populations. National AIDS Program of Sierra Leone.

[12] Ditmore MH (2014). Caught between the tiger and the crocodile: Cambodian sex workers’ experiences of structural and physical violence. Stud Gend Sex.

[13] Thukral J, Ditmore MH (2003). Revolving door. urban justice center sex workers project. https://swp.urbanjustice.org/wp-content/uploads/sites/14/2019/09/RevolvingDoor.pdf.

[14] Ditmore MH, Maternick A, Zapert K (2012). The Road North: The Role of Gender, Poverty and Violence in Trafficking from Mexico to the US.

[15] Schnall R, Rojas M, Bakken S (2016). A user-centered model for designing consumer mobile health (mHealth) applications (apps). J Biomed Inform.

[16] De Vito Dabbs A, Myers BA, Mc Curry KR (2009). User-centered design and interactive health technologies for patients. Comput Inform Nurs.

[17] Carr C, King LM, Maizel J (2024). Strategies and interventions used to prevent violence against sex workers in the United States: a scoping review using the social-ecological model. Trauma Violence Abuse.

[18] Lyon AR, Munson SA, Renn BN (2019). Use of human-centered design to improve implementation of evidence-based psychotherapies in low-resource communities: protocol for studies applying a framework to assess usability. JMIR Res Protoc.

[19] Graham AK, Munson SA, Reddy M (2021). Integrating user-centered design and behavioral science to design a mobile intervention for obesity and binge eating: mixed methods analysis. JMIR Form Res.

[20] Noergaard B, Sandvei M, Rottmann N (2017). Development of a web-based health care intervention for patients with heart disease: lessons learned from a participatory design study. JMIR Res Protoc.

[21] Cornet VP, Toscos T, Bolchini D (2020). Untold stories in user-centered design of mobile health: practical challenges and strategies learned from the design and evaluation of an app for older adults with heart failure. JMIR Mhealth Uhealth.

[22] Experience WL in RBU 10 usability heuristics for user interface design. https://www.nngroup.com/articles/ten-usability-heuristics/.

[23] Uribe-Ocampo S, Torres EA, Luna IF, Florez-Arango JF, Smith JW (2019). SIM-CIG: a serious game to practice and improve clinical guidelines adoption based on computer-interpretable guidelines. Stud Health Technol Inform.

[24] Zhang J, Wallji M (2011). TURF: Toward a unified framework of EHR usability. J Biomed Inform.

[25] Nielsen J (1994). Estimating the number of subjects needed for a thinking aloud test. Int J Hum-Comput Stud.

[26] Or C, Tao D (2012). Usability study of a computer-based self-management system for older adults with chronic diseases. JMIR Res Protoc.

[27] Brooke J (2023). SUS - A quick and dirty usability scale. https://www.usability.gov/how-to-and-tools/methods/system-usability-scale.html.

[28] Cunningham S, Sanders T, Scoular J (2018). Behind the screen: commercial sex, digital spaces and working online. Technol Soc.

[29] Campbell R, Sanders T, Scoular J, Pitcher J, Cunningham S (2019). Risking safety and rights: online sex work, crimes and ‘blended safety repertoires.’. Br J Sociol.

[30] Moorman JD, Harrison K (2016). Gender, race, and risk: intersectional risk management in the sale of sex online. J Sex Res.

[31] Al-Rawi A, Zemenchik K (2022). Sex workers’ lived experiences with COVID-19 on social media: content analysis of Twitter posts. JMIR Form Res.

