# Supplementary material for: User-Centered Design for Designing and Evaluating a Prototype of a Data Collection Tool to Submit Information About Incidents of Violence Against Sex Workers: Multiple Methods Approach
Source: JMIR Hum Factors. 2024 Oct 9;11:e53557. doi: 10.2196/53557 (PMC11481817; doi:10.2196/53557)
Supplement: Multimedia Appendix 1 [file humanfactors-v11-e53557-s001.docx]

Scenario 1. I work in a brothel and we were robbed. One person came as a client, others followed and were let in by the first. They had guns and took our money. One person was hit, then everyone cooperated and they left. It took only a few minutes that went more slowly than any other time in my life. One of them had come as a [client] before and knew some of the layout.

Scenario 2. Someone pretending to be a [client] picked me up on the street, and drove me far away from everything. He beat me up. I was punched, pulled out of the car, kicked and pulled my hair. I have a black eye and a fat lip. I broke my shoe running away.

Scenario 3. This one cop, if he sees me, he keeps me in the squadcar. He has forced me to blow him, but not every time.
